# The selective sigma-1 receptor antagonist E-52862 attenuates neuropathic pain of different aetiology in rats

**DOI:** 10.1038/srep24591

**Published:** 2016-04-18

**Authors:** Georgia Gris, Enrique Portillo-Salido, Bertrand Aubel, Yassine Darbaky, Kristof Deseure, José Miguel Vela, Manuel Merlos, Daniel Zamanillo

**Affiliations:** 1 Department of Pharmacology, Drug Discovery & Preclinical Development, ESTEVE, Barcelona, Spain; 2 ANS Biotech, RIOM Cedex, France.; 3 Laboratory of Anesthesiology, University of Antwerp, Antwerp, Belgium

## Abstract

E-52862 is a selective σ_1_R antagonist currently undergoing phase II clinical trials for neuropathic pain and represents a potential first-in-class analgesic. Here, we investigated the effect of single and repeated administration of E-52862 on different pain-related behaviours in several neuropathic pain models in rats: mechanical allodynia in cephalic (trigeminal) neuropathic pain following chronic constriction injury of the infraorbital nerve (IoN), mechanical hyperalgesia in streptozotocin (STZ)-induced diabetic polyneuropathy, and cold allodynia in oxaliplatin (OX)-induced polyneuropathy. Mechanical hypersensitivity induced after IoN surgery or STZ administration was reduced by acute treatment with E-52862 and morphine, but not by pregabalin. In the OX model, single administration of E-52862 reversed the hypersensitivity to cold stimuli similarly to 100 mg/kg of gabapentin. Interestingly, repeated E-52862 administration twice daily over 7 days did not induce pharmacodynamic tolerance but an increased antinociceptive effect in all three models. Additionally, as shown in the STZ and OX models, repeated daily treatment with E-52862 attenuated baseline pain behaviours, which supports a sustained modifying effect on underlying pain-generating mechanisms. These preclinical findings support a role for σ_1_R in neuropathic pain and extend the potential for the use of selective σ_1_R antagonists (e.g., E-52862) to the chronic treatment of cephalic and extra-cephalic neuropathic pain.

Neuropathic pain is characterized by spontaneous ongoing or shooting pain and evoked amplified pain responses after noxious or non-noxious stimuli^1^. The current therapy for neuropathic pain is not satisfactory and thus new drugs acting on new molecular targets are being investigated^2^,^3^. Several therapeutic approaches targeting different modulatory proteins have emerged. Among them, the sigma-1 receptor (σ_1_R) has been described to play a role in pain control^4^. σ_1_R is an intracellular chaperone protein that interacts with other proteins, including plasma membrane and endoplasmic reticulum receptors and ion channels. In the context of pain, σ_1_R modulates central sensitization phenomena^5^,^6^, which are responsible for many of the temporal, spatial, and threshold changes in pain sensitivity in acute and chronic pain^7^. Accordingly, pharmacological treatment with σ_1_R antagonists in wild-type (WT) mice exerted antinociceptive effects and σ_1_R knockout (KO) mice showed a pain-reduced phenotype in different experimental pain models^6^,^8^,^9^,^10^,^11^,^12^,^13^,^14^,^15^.

The *in vitro* and *in vivo* pharmacological profile of the σ_1_R antagonist E-52862 (S1RA) has been described^6^. E-52862 shows high σ_1_R affinity and selectivity. It binds to σ_1_R in the CNS when administered systemically, as shown by autoradiographic *ex vivo* binding assays in mice, and its efficacy correlates with the occupancy of σ_1_Rs. It shows a good preclinical safety and efficacy profile in mice^6^. Specifically, formalin-induced nociception^6^, capsaicin-induced mechanical allodynia^6^, paclitaxel-induced cold and mechanical allodynia^15^, nerve injury-induced mechanical and thermal hypersensitivity^6^ and inflammation-induced mechanical and thermal hypersensitivity^13^,^14^ were dose-dependently inhibited by acute systemic administration of E-52862.

E-52862 has completed single- and multiple-dose phase I clinical studies demonstrating good safety, tolerability and pharmacokinetic profiles in humans^16^, and is currently in phase II clinical trials for the treatment of neuropathic pain of different aetiology using a daily oral dose of 400 mg. In the present study, we tested the efficacy of E-52862 in three rat models of neuropathic pain of different aetiologies: trigeminal neuropathic pain following chronic constriction injury to the infraorbital nerve (IoN)^17^, streptozotocin (STZ)-induced diabetic neuropathy^18^, and oxaliplatin (OX)-induced painful neuropathy^19^. These neuropathic pain models simulate clinical pain conditions with diverse aetiologies, such as trigeminal neuralgia^20^, diabetic painful polyneuropathy^21^, and chemotherapy-induced neuropathic pain^22^. As neuropathic pain is a persistent (chronic) type of pain which, in clinical practice, frequently requires long-term pharmacological treatments, E-52862 was repeatedly administered to neuropathic rats for several days, and its chronic analgesic effects were compared with the acute effects.

## Results

### Development of mechanical allodynia in the neuropathic pain model of constriction injury of the infraorbital nerve (IoN)

**Figure 1:**
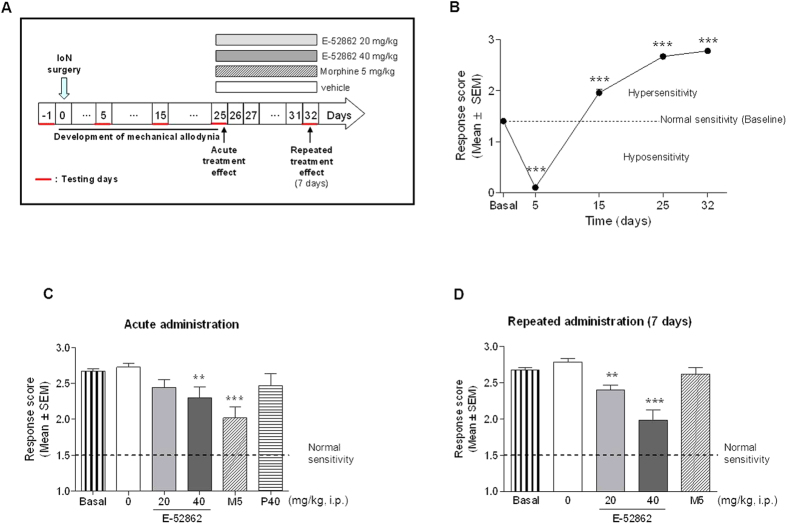
Trigeminal neuropathic pain model of chronic constriction injury of the IoN. (**A**) Experimental protocol. The acute treatment effects of the drugs were evaluated on day 25 after IoN surgery. The repeated administrations of the drugs were evaluated on day 32 after 7 days of repeated daily administration. (**B**) Time-related course of mechanical allodynia after IoN ligation. Evaluation was performed one day before surgery (baseline) and on postoperative days 5, 15 and 25 (black circles). (**C**) Effect of a single i.p. administration of E-52862 (20 and 40 mg/kg, i.p.; grey bars), morphine (5 mg/kg; diagonal dashed bars) and pregabalin (40 mg/kg; horizontal dashed bars) on mechanical allodynia. (**D**) Effect of repeated i.p. administration of E-52862 (20 and 40 mg/kg; grey bars) and morphine (5 mg/kg; diagonal dashed bars) on mechanical allodynia. Each point and vertical line represent the mean ± S.E.M. of the values obtained in at least 10 rats per treatment and baseline groups. ***p < 0.001 *vs*. pre-surgery in (**B**). **p < 0.01; ***p < 0.001 *vs*. vehicle group in (**C**) and (**D**) (one-way ANOVA followed by Newman–Keuls test).

Baseline values were obtained one day before surgery, setting the normal response to von Frey filaments (Fig. 1A). Chronic constriction of the IoN induced significant changes in response to mechanical stimulation of the territory innervated by the ligated ipsilateral IoN (Fig. 1B). Initially, 5 days after surgery, the response score dropped significantly, indicating hyposensitivity, but this was followed by a robust hypersensitivity to von Frey filament stimulation on days 15 and 25 after IoN surgery, and hypersensitivity was maintained at least for 32 days after IoN constriction (F_4,233_ = 533.7, *p* < 0.001, ANOVA; *p* < 0.001 for days 5, 15, 25 and 32 *vs*. baseline).

### Effect of acute administration of E-52862, morphine and pregabalin on mechanical allodynia developed after chronic constriction injury of the IoN

Drugs were administered when mechanical allodynia fully developed, on day 25 post-surgery. Single i.p. administration of E-52862 significantly inhibited mechanical allodynia at 40 mg/kg, but not at 20 mg/kg. Similarly, morphine (5 mg/kg) exerted an antiallodynic effect (F_5,124_ = 8.5, *p* < 0.001, ANOVA; *p* < 0.01 for E-52862 40 mg/kg *vs*. vehicle; *p* < 0.001 for morphine 5 mg/kg *vs*. vehicle) but pregabalin (40 mg/kg) was ineffective (Fig. 1C).

### Effect of repeated administration of E-52862 and morphine on mechanical allodynia developed after chronic constriction injury of the IoN

To evaluate the effect of repeated administrations, E-52862 and morphine were administered b.i.d. for 7 days (from day 25 to day 32 post-surgery) (Fig. 1A). After 7 days of administration, E-52862 exerted antinociceptive effect (antiallodynic; reduction of the response score) at both 20 and 40 mg/kg. In contrast, morphine (5 mg/kg) developed tolerance, as it was devoid of antinociceptive effects when assayed after repeated administration for 7 days (F_3,49_ = 17.4, *p* < 0.001, ANOVA; *p* < 0.001 and *p* < 0.01 for E-52862 40 and 20 mg/kg, respectively, *vs*. vehicle).

### Development of mechanical hyperalgesia in the STZ-induced neuropathic pain model

**Figure 2:**
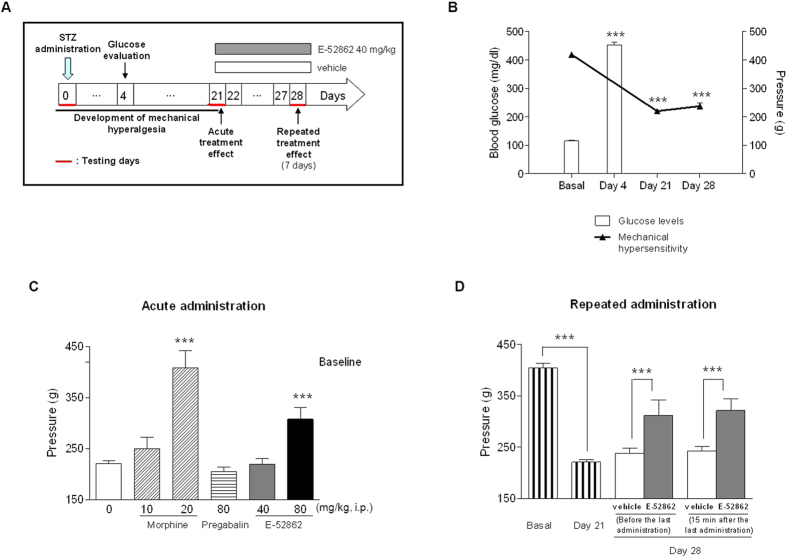
Diabetic neuropathic pain model after STZ injection. (**A**) Experimental protocol. The acute treatment effects of the drugs were evaluated on day 21 after the STZ injection. The repeated administrations of the drugs were evaluated on day 28 after 7 days of daily administration. (**B**) Time-related course of mechanical hyperalgesia after STZ injection (black triangles) and blood glucose levels (white bars) evaluated 4 days post-STZ injection. (**C**) Effects of a single i.p. administration of E-52862 (40 and 80 mg/kg; grey and black bars), morphine (10 and 20 mg/kg; diagonal dashed bars) and pregabalin (80 mg/kg; horizontal dashed bars) on mechanical hyperalgesia. (**D**) Effect of repeated i.p. administration of E-52862 (40 mg/kg; grey bars) on mechanical hyperalgesia. Rats were evaluated before and 15 min after the last administration. Each point and vertical line represents the mean ± S.E.M. of the values obtained from at least 8 animals per treatment group. Difference between pre- and post-STZ in glucose levels and mechanical hyperalgesia: ***p < 0.001 (Student’s t-test for glucose values and one-way ANOVA followed by Newman–Keuls test for hyperalgesia development of); ***p < 0.001 *vs*. vehicle group (HPMC 0.5%) (one-way ANOVA followed by Newman–Keuls test).

Rats showed increased glucose levels 4 days after STZ injection (453.3 ± 9.0 mg/dl *vs*. 115.7 ± 1.8 mg/dl, *p* < 0.001; Fig. 2B) and exhibited a robust reduction of paw withdrawal thresholds in response to noxious pressure stimulation (mechanical hyperalgesia) by 3-4 weeks after the injection of STZ (from 418.9 ± 5.4 to 220 ± 5.6 grams, F_2,162_ = 354.4, *p* < 0.001, ANOVA; *p* < 0.001 for days 21 and 28 post-STZ *vs*. baseline) (Fig. 2B).

### Effect of acute administration of E-52862, morphine and pregabalin on mechanical hyperalgesia in the STZ-induced neuropathic pain model

Systemic acute administration of 80 mg/kg —but not 40 mg/kg— of E-52862 significantly decreased mechanical hypersensitivity in STZ-treated rats by 44% (F_6,122_ = 20.9, *p* < 0.001, ANOVA; *p* < 0.001 for E-52862 80 mg/kg *vs*. vehicle; Fig. 2C). Morphine at 20 mg/kg reversed mechanical hypersensitivity in diabetic rats. In contrast, morphine at 10 mg/kg and pregabalin at 80 mg/kg failed to show any significant effect (Fig. 2C).

### Effect of repeated administration of E-52862 on mechanical hyperalgesia developed in the STZ-induced neuropathic pain model

The effect of repeated dosing b.i.d. for 7 consecutive days (from day 21 to day 27 after STZ injection) with 40 mg/kg of E-52862 (cumulative dose per day 80 mg/kg) on mechanical hypersensitivity developed in STZ-treated rats was evaluated (Fig. 2D). As expected, STZ administration induced hyperalgesia to mechanical stimulation of hind paws (F_7,112_ = 37.4, *p* < 0.001, ANOVA; *p* < 0.001 for baseline *vs*. day 21). On day 28, the day after completion of the 7-day treatment period (16 hours after the last E-52862 administration) and before treatment (pre-dose), mechanical hypersensitivity was decreased in STZ-treated rats that received E-52862 (from 237.8 ± 10.5 g in HPMC-treated rats to 311.7 ± 30.8 g in E-52862-treated rats). The administration of one additional dose of 40 mg/kg of E-52862 on day 28 (post-dose) failed to significantly change the mechanical threshold respect to the pre-dose value (hyperalgesia was reduced as compared to 7-day vehicle-treated rats and similar to pre-dose, but additional dosing was unable to further reduce hyperalgesia) (F_7,112_ = 37.4, *p* < 0.001, ANOVA; *p* < 0.001 for E-52862 40 mg/kg *vs*. HPMC at both pre- and post-dose assessments on day 28).

### Development of cold allodynia in the OX-induced neuropathic pain model

**Figure 3:**
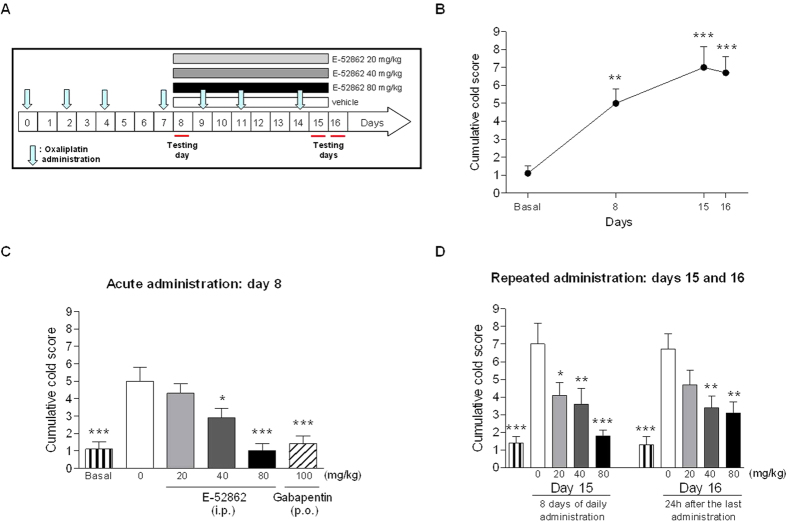
Chemotherapy-induced neuropathic pain after OX treatment. (**A**) Experimental protocol. The acute treatment effects of the drugs were evaluated on day 8 after four OX administrations. The repeated administration of drugs was evaluated on day 15 after 7 days of daily administration. The cumulative cold scores in response to acetone were evaluated on days 8, 15 and 16 after the baseline reading (testing days). (**B**) Time-related course of cold allodynia after OX treatment evaluated as cumulative cold score. (**C**) Effects of a single i.p. administration of E-52862 (grey and black bars) and gabapentin (diagonal dashed bars) at 40 and 100 mg/kg, respectively, on cold allodynia. (**D**) Dose-response effects of repeated administration of E-52862 (20, 40 and 80 mg/kg) and response to cold stimulus one day after the last administration (day 16). Each point and vertical line represents the mean ± S.E.M. of the values obtained from 10 animals per group. *p < 0.05; **p < 0.01; ***p < 0.001 *vs*. each vehicle group (one-way ANOVA followed by Newman–Keuls test).

OX administration (Fig. 3A) induced cold allodynia, i.e., increased cumulative cold score in the acetone test respect to HPMC (vehicle)-treated animals on days 8, 15 and 16 (F_3,36_ = 10.1, *p* < 0.001 for the cumulative cold score; Fig. 3B).

### Effect of acute administration of E-52862 and gabapentin on cold allodynia in the OX-induced neuropathic pain model

Neuropathy-related cold allodynia was already statistically significant as compared to the non-neuropathic group on day 8 after initiating OX administration (1.1 ± 0.4 *vs*. 5.0 ± 0.8, respectively; *p* < 0.01 and *p* < 0.001; Fig. 3B). In the acute treatment experiments (day 8), E-52862 exerted a dose-dependent antiallodynic effect on the cumulative cold score (Fig. 3C). The lowest tested dose (20 mg/kg) did not significantly reduce cold allodynia but E-52862 exerted a significant antinociceptive effect when administered at 40 mg/kg (F_5,54_ = 10.2, *p* < 0.001, ANOVA; *p* < 0.05 for E-52862 40 mg/kg *vs*. vehicle) and reversed cold allodynia back to normal baseline values at 80 mg/kg. As standard analgesic drug, the acute effect of gabapentin was also evaluated. Gabapentin orally administered at 100 mg/kg on day 8 reversed OX-induced cold allodynia as evidenced by a significantly decreased cumulative score in the acetone test.

### Effect of repeated administration of E-52862 on the expression of cold allodynia in the OX-induced neuropathic pain model

To evaluate the effect of repeated administration, E-52862 was dosed from day 8 to 15 (Fig. 3A). On day 15 (last day of administration), cold allodynia was significantly inhibited by E-52862 at all three doses (20, 40 and 80 mg/kg) and in a dose-dependent manner (F_4,45_ = 8.5, *p* < 0.001, ANOVA; *p* < 0.001, *p* < 0.01 and *p* < 0.05 for E-52862 at 80, 40 and 20 mg/kg, respectively, *vs*. vehicle; Fig. 3D). Interestingly, reduced cold allodynia was still noticeable after treatment completion on day 16, 24 h after last administration (F_4,45_ = 8.0, *p* < 0.001, ANOVA; *p* < 0.01 for E-52862 at 40 and 80 mg/kg *vs*. vehicle; Fig. 3D).

### Effect of repeated administration of E-52862 on the development of cold allodynia in the OX-induced neuropathic pain model (preventive protocol)

Unlike other neuropathic pain conditions, chemotherapy administration to cancer patients is a planned/scheduled procedure and thus neuropathic pain can be awaited (and anticipated) in a short term frame following administration of the cytostatic. Thus, it makes sense in this case to investigate preventive approaches that could attenuate this undesired effect of antineoplastics.

**Figure 4:**
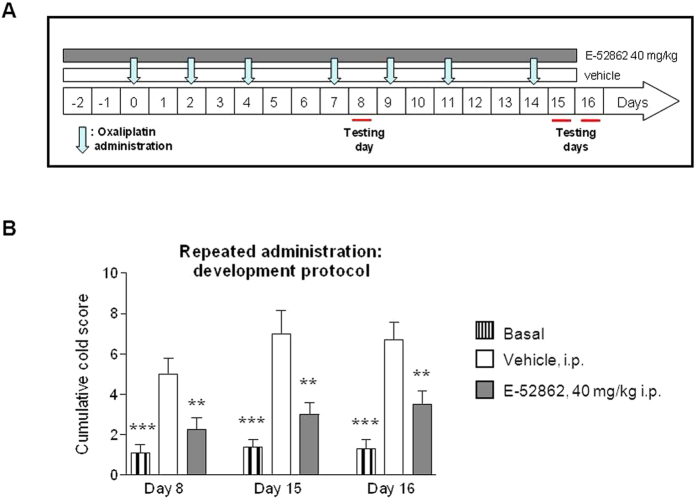
Effect of repeated administration of E-52862 on cold allodynia in a preventive paradigm in OX-induced neuropathy in rats. (**A**) Experimental preventive protocol. In this case, the administration of E-52862 started two days before the first OX injection. The preventive effect was evaluated on days 8, 15 and 16 after the first injection of OX (testing days). The evaluation on day 16 was performed one day after the last administration of E-52862. (**B**) Effects of the repeated administration of E-52862 at 40 mg/kg (grey bars) in the preventive protocol on cold allodynia evaluated as cumulative cold score. Each point and vertical line represents the mean ± S.E.M. of the values obtained from 10 animals per group. **p < 0.01; ***p < 0.001 *vs*. vehicle group (one-way ANOVA followed by Newman–Keuls test).

Therefore, given the potential benefit of co-administering patients with antineoplastic drugs and drugs counteracting antineoplastic’s undesired effects, the possible preventive effect of E-52862 on the development of OX-induced neuropathic pain (i.e., cold allodynia) was also investigated. For this purpose, E-52862 was administered i.p. b.i.d at 40 mg/kg starting 2 days before the first injection of OX and throughout the OX treatment period (last OX injection on day 14) (Fig. 4A). On day 8, rats co-treated with OX and E-52862 b.i.d. at 40 mg/kg exhibited significantly lower cumulative cold scores as compared to the OX + vehicle group (*p* < 0.01 for comparisons between E-52862 40 mg/kg *vs*. vehicle; Fig. 4B). One week later, on day 15, the antiallodynic effect of the treatment was maintained (F_2,25_ = 13.5, *p* < 0.001, ANOVA; *p* < 0.01 E-52862 40 mg/kg *vs*. vehicle). Interestingly, 24 hours after E-52862 treatment completion (day 16) animals showed reduced cold allodynia (F_2,25_ = 16.4, *p* < 0.001, ANOVA; *p* < 0.01 E-52862 40 mg/kg *vs*. vehicle) compared to the vehicle-treated OX-treated group (3.5 ± 0.7 *vs*. 6.7 ± 0.9), although cumulative pain scores were higher than baseline values in non-neuropathic animals that did not receive OX (F_2,25_ = 16.4, *p* < 0.001, ANOVA; *p* < 0.05 for comparisons between E-52862 40 mg/kg *vs*. baseline).

## Discussion

A rather compelling role of σ_1_R in pain has been proposed from preclinical studies in models of different types of pain, but most studies have used mice as experimental subjects, peripheral nerve surgery as experimental model of neuropathy and acute treatment approaches. In the present study, these findings were broadened by using rats in three different models of neuropathic pain with translational value to measure disease-related pain processes of diverse aetiology and huge unmet need for treatment. In this sense, trigeminal neuralgia^20^, diabetic painful polyneuropathy^21^, and chemotherapy-induced neuropathic pain^22^ are important clinical pain conditions often refractory to current pharmacotherapies. Not only single (acute) but also repeated (subchronic/chronic) treatment with E-52862^6^, a selective σ_1_R antagonist, was investigated to find out if sustained pharmacological blockade of σ_1_R induces tolerance and if repeated administrations could interfere with the expression of neuropathic pain. Different evoked mechanical and thermal readouts were measured to monitor pain development and the antinociceptive effect of drug treatments. The main findings were that E-52862 exerted antinociceptive effects across the different models of neuropathic pain in rats and that its effects were not only maintained but increased following repeated administration. The present data extend recent evidence that σ_1_R antagonism constitutes a new mechanism of analgesia the spectrum of which may also encompass chronic treatment of both cephalic (trigeminal) and extra-cephalic neuropathic pain.

Most neuropathic pain models are actually models of extra-cephalic neuropathic pain as they rely on the injury of spinal nerves with primary relay involving neurons at the dorsal root ganglia and the dorsal horn of the spinal cord. As opposed to the extra-cephalic one, cephalic neuropathic pain affects cranial nerve territories and involves primary synaptic relay by neurons at cephalic ganglia and brainstem nuclei. Not only the anatomy but also the pathophysiology and the response to analgesics differ when comparing extra-cephalic and cephalic neuropathic pain, both preclinically and clinically^23^,^24^,^25^,^26^. The rat model of chronic constriction injury of the fifth cranial nerve (infraorbital nerve; IoN) has been reported to be a model for cephalic (trigeminal) neuropathic pain (i.e., trigeminal neuralgia) in humans^25^,^26^,^27^. As shown in previous studies^17^,^28^, we found that nociceptive behaviours following IoN injury were characterized by a robust mechanical hypersensitivity preceded by a transient phase of lower responsiveness to mechanical stimulation of the IoN territory. Acute administration of morphine and E-52862 —but not pregabalin— reversed mechanical allodynia observed in this pain model. The acute antinociceptive effect of morphine and the lack of efficacy of gabapentin (another gabapentinoid similar to pregabalin) had been previously reported^29^,^30^. The antinociceptive effect of E-52862 was the first evidence of efficacy with a σ_1_R ligand in this cephalic pain model, where gabapentinoids —that work in extra-cephalic neuropathic pain models— are ineffective but antimigraine drugs such as triptans (sumatriptan and zolmitriptan), dihydroergotamine and olcegepant —that are essentially inactive in extra-cephalic pain— are effective^23^,^26^. The efficacy of E-52862 in the IoN model is in agreement with some recent literature showing acute inhibitory effects of the σ_1_R antagonist BD1047 on the nociceptive activation of the trigeminal nucleus caudalis in the capsaicin-induced headache model in rats^31^ and the behavioural nociceptive responses in the orofacial formalin model in mice^32^. In turn, activation of σ_1_R by intracisternal administration of the σ_1_R agonist PRE084 evoked nociceptive activation of trigeminal nucleus caudalis in rats, which the antagonist BD1047 reversed^33^. Finally, it is important to note that administration of the σ_1_R antagonist E-52862 for 7 days did not induce antinociceptive tolerance in a protocol where tolerance to the antinociceptive effect of morphine was clearly seen. On the contrary, the effect was increased when preceded by previous administrations as efficacy following repeated dosing was significant at 20 mg/kg (the lower dose used), a dose that was insufficient to elicit significant antiallodynic effects after single acute administration.

Regarding extra-cephalic neuropathic pain, diabetic neuropathy is amongst the most frequent long-term complications of diabetes mellitus, and current treatments are only partially effective. STZ model in rats was selected as it is the most widely used model for the study of the diabetic painful polyneuropathy in rodents, where many standard analgesics have been tested^34^,^35^,^36^. In agreement with previous reports^36^,^37^, rats injected with this toxin for pancreatic β-cells exhibited significantly increased plasma glucose and water intake, decreased body weight, and increased sensitivity to noxious pressure stimuli (i.e., mechanical hyperalgesia) as compared to control, non-diabetic rats, thus reproducing symptoms observed in diabetic humans^38^,^39^. In addition to E-52862, two marketed drugs (morphine and pregabalin) with different mechanism of action were used to treat mechanical hypersensitivity of STZ-treated rats. Single administration of morphine at 10 mg/kg (a rather high dose) did not produce any significant effect but morphine reversed mechanical hyperalgesia when administered at 20 mg/kg (a very high dose). This result adds to the evidence that sensitivity to the analgesic effect of morphine is low in diabetic animals^40^,^41^, and it is consistent with clinical reports that morphine lacks efficacy for the symptomatic relief of neuropathic pain in diabetic patients^42^,^43^. Similarly, the administration of a quite high dose of pregabalin (80 mg/kg) was ineffective in our model. In this sense, pregabalin has shown to provide some degree of pain relief in patients with painful diabetic neuropathy^44^, and other studies reported significant antinociceptive effects of this drug on STZ-induced diabetic rats; however, this effect was modest when mechanical hyperalgesia was evaluated^45^. Single administration of E-52862 significantly reduced mechanical hypersensitivity in STZ-treated rats at the dose of 80 mg/kg, but not at 40 mg/kg. This is the first reporting of efficacy of a σ_1_R ligand in a model of diabetic neuropathy and thus cannot be discussed against findings in other studies, but it is consistent with the antihyperalgesic effects exerted by E-52862 and other σ_1_R antagonists^4^,^6^,^13^,^46^ and the “pain-resistant” phenotype of σ_1_R KO mice in a spectrum of pain conditions^9^,^11^,^12^,^15^. Just to note that STZ-induced mechanical hyperalgesia seems to be a difficult to treat pain condition, at least in our hands, based on both the lack of efficacy/reduced potency of marketed drugs and the activity of E-52862, lower than in other models/readouts, including the previously described IoN model. Interestingly, the dose of 40 mg/kg, that failed to produce any significant effect in the acute treatment, was effective against mechanical hypersensitivity in diabetic rats when preceded by repeated E-52862 40 mg/kg administration b.i.d. for 7 consecutive days. In fact, hyperalgesia was attenuated already before the last administration (previous injection of 40 mg/kg of E-52862 the day before), which supports not only lack of tolerance but suggests a modifying effect on the underlying baseline pain (or alternatively drug accumulation, as discussed later).

A third model, the OX-induced neuropathy model in rats was used to mimic neuropathic pain observed in cancer patients receiving this chemotherapy agent^19^,^47^. OX is a third-generation platinum-based antineoplastic drug used for the treatment of colorectal cancer that induces painful neuropathy characterized by marked cold sensitivity^19^,^48^. Consistent with clinical symptoms, OX-treated rats showed cold allodynia already by day 8 after initiating OX treatment. Other authors using several rodent models of chemotherapy-induced neuropathy have found similar results^49^,^50^. Single oral administration of 100 mg/kg of gabapentin on day 8 reduced this pain-related behaviour, which is in accordance with previous findings in the same neuropathic pain model^19^,^50^,^51^. Gabapentin is a well-known gabapentinoid drug with the same mechanism of action of pregabalin. They are both amino acid derivative of gamma-amino butyric acid (GABA analogue) and have similar pharmacological profile^52^. Regarding σ_1_R modulation, E-52862 reduced cold allodynia. The effect of acute administration of E-52862 on day 8 was dose-dependent and equivalent in efficacy to gabapentin. Again, this is the first study describing efficacy of a σ_1_R ligand in this model, but in this case our data are supported by findings in a different chemotherapy model and species, the paclitaxel-induced neuropathic pain model in mice. Paclitaxel-induced mechanical and cold allodynia was dose-dependently reverted by E-52862 and BD1063, another σ_1_R antagonist, and it did not develop in σ_1_R KO mice, which reinforces pharmacological data with the antagonists^15^,^53^. In this way, it is important to note that antineoplastic drugs produce a painful peripheral neuropathy characterized by mitochondrial alterations in peripheral nerves, that prophylactic treatment of mice with BD1063 prevented not only paclitaxel-induced allodynia but also mitochondrial alterations, and that paclitaxel treatment did not induce mitochondrial abnormalities in σ_1_R KO mice^53^. Moreover, as in the two previous models, no tolerance but increased activity respect to the acute treatment was found following repeated E-52862 administration in the OX model (e.g. the dose of 20 mg/kg, ineffective after single treatment, exerted significant antiallodynic effect on day 15, the last day of treatment, when preceded by repeated E-52862 administration for 7 consecutive days). Also, similar to findings in the STZ model, the pain-related behaviour was attenuated in the OX model one day after the last administration of E-52862 (day 16; 24 h washout, in the absence of active treatment) both when administered from day 8 for 7 consecutive days and when co-administered with OX throughout the OX treatment period (E-52862 administered i.p. b.i.d at 40 mg/kg starting 2 days before the first injection of OX and until the last OX injection on day 15), which again suggests a modifying effect on the underlying baseline pain (or alternatively drug accumulation, as discussed below).

Repeated treatments with E-52862 resulted in higher efficacy and potency respect to single/acute treatments consistently in all three neuropathic pain models. That is, higher antinociceptive activity and lower doses were required to reduce the different pain-related behaviours if the administration the day of the test was preceded by repeated E-52862 daily (7 days, b.i.d.) administrations. This discards pharmacodynamic tolerance phenomena but opens the possibility that repeated treatments could exert a modifying effect on baseline pain (i.e., a sustained pharmacodynamic effect on nociceptive thresholds over time due to the continued action of the compound that results in a progressive attenuation of pain). The observation that pain-related behaviours were reduced the day after repeated treatment completion in E-52862-treated respect vehicle-treated animals further support the possibility of a sustained pharmacodynamic effect. Alternatively, increased antinociceptive effects following repeated administration could be explained by a pharmacokinetic effect due to drug accumulation over time. However, this is highly unlikely due to the pharmacokinetic profile of E-52862 in rodents. The maximum plasma concentration is achieved shortly after its administration to rodents (t_max_ = 15 min after i.p. administration to mice and rats) and is quickly metabolized, having a short half-life (t_1/2_ = 1.4 h after administration to mice and rats). Undetectable plasma levels were found by 6 h after its administration, its metabolites are inactive and it does not accumulate in tissues, including the brain and the spinal cord^6^. All together, pharmacodynamics, but not phamacokinetics (i.e., drug accumulation), can explain the increased antinociceptive effect after repeated administration of E-52862 and also the attenuated hyperalgesia and allodynia found without E-52862 administration the day after treatment completion. Interestingly, it is in line with the mechanism of action of σ_1_R and the inhibitory effect attributed to σ_1_R antagonism on central sensitization phenomena, as reported at the behavioural, electrophysiological and molecular level. Behaviourally, it is known that a) capsaicin-induced secondary mechanical hypersensitivity (e.g., allodynia) results from central sensitization (i.e., plastic changes at the spinal cord due to the initial intense nociceptive discharges that follows the capsaicin injection and result in subsequent increased pain sensitivity), that b) σ_1_R antagonists block capsaicin-induced mechanical allodynia^6^,^10^,^11^; and that c) capsaicin does not induce mechanical allodynia when injected to σ_1_R KO mice^11^. Electrophysiologically, it is known that repetitive stimulation of the dorsal root at stimulus intensities activating C fibres produces a typical amplification of the nociceptive signals in the spinal cord (wind-up response). Wind-up is the result of the sensitization of spinal dorsal horn neurons whose increased excitability is evoked by repetitive stimulation of afferent C fibres, and stands for a spinal amplification of the message coming from peripheral nociceptors^54^,^55^,^56^. σ_1_R antagonists (i.e., E-52862) dose-dependently inhibit the spinal wind-up phenomenon when trains of nociceptive stimuli (repetitive stimulation of nociceptive afferent fibres) were applied^6^,^57^ and, accordingly, spinal wind-up amplification of the nociceptive signals is highly reduced in spinal cords from σ_1_R KO mice^12^. Finally, at the molecular level, increased signalling through the glutamatergic NMDA receptor on dorsal horn neurons is known to be a key mediator of spinal wind-up^56^,^58^, and account for sensitization and pain hypersensitivity^7^. The NMDA receptor itself becomes phosphorylated in dorsal horn neurons following noxious stimulation or nerve injury, and this facilitates NMDA responses and thus central sensitization and pain hypersensitivity. Ligands of σ_1_R are postsynaptic regulators of NMDA receptor-mediated synaptic transmission. Activation of σ_1_R enhances the NMDA receptor-mediated rise in cytosolic Ca^2+^ concentration and currents^59^,^60^. In contrast, the NMDA receptor-mediated responses are inhibited and the enhancing effects of σ_1_R agonists on NMDA receptors blocked by antagonizing σ_1_R^59^,^61^. Accordingly, activation of spinal σ_1_R by intrathecal administration of the σ_1_R agonist PRE084 evoked pain concomitant with increased phosphorylation of the NMDA receptor NR1 subunit, and both nociceptive behaviours and increased phosphorylation of NR1 in the spinal cord were inhibited by antagonizing spinal σ_1_R with BD1047^62^,^63^,^64^. From the mechanistic point of view, we now know that σ_1_R interact with the NR1 subunit of NMDA receptors and that σ_1_R antagonists (E-52862) remove the binding of σ_1_R to NR1 subunits, facilitating the entrance of negative regulators of NMDA receptor activity, likely Ca^2+^/calmodulin, which results in reduced glutamate-dependent NMDA receptor-mediated pain signalling and amplification^65^. Overall, evidence supports a role for σ_1_R in modulating nociception by inhibiting augmented excitability secondary to sustained afferent drive as a mechanism underlying its modulatory effect. Attenuation of plastic changes (central sensitization) following nerve injury could thus underlie the sustained pharmacodynamic modifying effect on pain hypersensitivity exerted by E-52862 following its repeated administration.

In summary, preclinical findings herein support a role of σ_1_R in both the expression and development of neuropathic pain and extend the potential use of σ_1_R antagonists (e.g., E-52862) for the chronic treatment of both cephalic (trigeminal) and extra-cephalic neuropathic pain, thus supporting progress to further studies in human populations. In fact, phase 2 clinical studies with E-52862 (400 mg, daily oral dose) in neuropathic pain of different aetiology (including from diabetic and chemotherapy origins) are ongoing.

## Methods

### Animals

Adult male Sprague-Dawley rats (IoN and OX experiments) and Wistar rats (STZ experiments) weighing between 150–250 g at the beginning of the experimental phase were used. Animals were provided with food and water *ad libitum* and kept in controlled laboratory conditions with the temperature maintained at 21 ± 1 °C and 12-hour light cycles (reversed dark/light cycle in IoN experiments, lights on at 20 h). Experiments were carried out in a soundproof and air-regulated experimental room. All experimental procedures and animal husbandry were conducted according to the ethical principles of the I.A.S.P. for the evaluation of pain in conscious animals^66^ and the European Parliament and the Council Directive of 22 September 2010 (2010/63/EU), and were approved by the Animal Ethics Committee of the University of Antwerp (IoN experiments), the Parc Cientific of Barcelona (STZ experiments) and the Facultés de Médecine et Phamacie of the University of Auvergne (OX experiments).

### Drugs

Oxaliplatin (OX) was provided by Shan Dong Boyuan Chemical Co, dissolved in distilled water and administered by intraperitoneal (i.p.) route. Streptozotocin (STZ) and acetone were provided by Sigma Aldrich. STZ was dissolved in 0.9% saline solution and administered by i.p. route. All analgesic drugs, except gabapentin, were administered i.p. Gabapentin was provided by Zhejiang Chiral Medicine Chemicals (China) and was administered at 100 mg/kg by oral (p.o.) route. E-52862 was synthesized by Laboratories Esteve (Spain), pregabalin by Mercachem (The Netherlands), and morphine was provided by the General Directorate of Pharmacy and Drugs (Spanish Ministry of Health; Madrid, Spain). Drugs were dissolved in saline, 0.5% hydroxypropyl methylcellulose (HPMC; Sigma Aldrich) or carboxymethylcellulose (CMC; Sigma Aldrich) as indicated. All drugs were administered at a volume of 10 ml/kg except in the IoN experiments —volume used 5 ml/kg.

The doses used for E-52862 were in the range of 20–80 mg/kg by intraperitoneal route which is the common administration route to investigate the antinociceptive effect of E-52862 in preclinical models^6^,^13^,^67^. These doses provide exposures (measured as maximum concentration, C_max_) corresponding to those of human oral doses of around 100–400 mg, p.o., being 400 mg the dose selected in the current Phase II clinical trials^16^.

The pre-treatment time for E-52862 to assess the antinociceptive effect was 15 or 30 min based on the pharmacokinetic studies of E-52862, which revealed maximal exposure 15–30 minutes following its administration in rodents.

### Experimental models

#### Chronic constriction of the infraorbital nerve (IoN)-induced trigeminal neuropathy

Surgical procedure: The infraorbital part of the nerve was exposed and ligated as described by Vos *et al*.^17^. Briefly, rats were anesthetized with pentobarbital (Nembutal, 60 mg/ml) at 60 mg/kg, i.p. followed by a fixed dose of 0.1 mg/kg atropine. The head of the rat was fixed in a stereotaxic frame and a mid-line scalp incision was made, exposing skull and nasal bone. The IoN was exposed using a surgical procedure similar to that described previously^68^,^69^. The edge of the orbit, formed by the maxillary, frontal, lacrimal, and zygomatic bones, was dissected free. The orbital contents were gently deflected with a cotton-tipped wooden rod, thus the IoN was dissected free at its most rostral extent in the orbital cavity. Two chronic catgut ligatures (5-0, Ethicon; Johnson and Johnson, Belgium) were loosely tied around the IoN (2 mm apart). To obtain the desired degree of constriction, a criterion proposed by Bennet and Xie^70^ was applied: the ligatures reduced the diameter of the nerve by a just noticeable amount and slowed but did not interrupt the circulation through the superficial vasculature. The scalp incision was closed using polyester sutures (4-0, Ethicon; Johnson and Johnson). In sham-operated rats, the IoN was exposed on one side using the same procedure, but the exposed IoN was not ligated.

#### Evaluation of mechanical allodynia

The responsiveness to mechanical stimulation of the IoN territory was measured using a series of five von Frey hairs (Stoelting Co): 0.015 g, 0.127 g, 0.217 g, 0.745 g and 2.150 g. Following a 10-min habituation, the different von Frey hairs were applied at every designated time to the ipsilateral side of the IoN territory. A mean score for the filaments was determined. Baseline data were obtained one day before surgery and on postoperative days 5, 15 and 25 (Fig. 1A). The scoring system described by Vos *et al*.^17^ was used to evaluate the reaction of the rats to the stimulation. The response of an animal was analyzed according to different response categories: 0: no response, 1: detection, 2: withdrawal reaction, 3: escape/attack and 4: asymmetric face grooming. Lower scores indicated a weak responsiveness to mechanical stimulation, whilst higher scores indicated a strong responsiveness. Rats were injected i.p. twice daily (b.i.d.) with compounds dissolved in 0.5% CMC. Behavioural testing was performed 30 min following injection.

#### Streptozotocin-induced diabetic neuropathy

Development of diabetes: Diabetes was induced in rats through chemical pancreatectomy by a single i.p. injection of STZ (75 mg/kg body weight) dissolved in saline, whereas control rats received saline alone^34^. Blood glucose levels were assessed before behavioural testing. Blood was extracted from the tail vein four days after STZ administration to calculate blood sugar levels by means of glucometer (Cholestech LDX). Upon diabetes induction (blood sugar levels above 240 mg/dl), food and water consumption, urinary volume and glucose levels increased in diabetic *versus* normal animals.

#### Evaluation of mechanical hyperalgesia

Mechanical hyperalgesia threshold was quantified using the Randall-Selitto test^71^ by means of a commercially available apparatus (Ugo Basile, Italy). The response to increasing pressure applied on the dorsum of the animal’s hind paw was measured. The nociceptive threshold was defined as the pressure, in grams, at which the rat withdraws its paw.

The mechanical sensitivity of naïve rats was first tested before STZ treatments and again three weeks after STZ administration in both left and right hind paws in order to select those animals showing a nociceptive mechanical threshold lower than 270 g. Pharmacological treatments were initiated 21 days after STZ injection, immediately after baseline determination, with vehicle (0.5% HPMC), morphine (10 and 20 mg/kg), pregabalin (80 mg/kg) and E-52862 (40 and 80 mg/kg) by i.p. route (Fig. 2A). Mechanical hyperalgesia was assessed 1 hour and 15 min after reference compounds and E-52862 administration, respectively. To determine the effects of repeated dosing on the hypersensitivity developed by STZ-treated rats, separate groups of STZ-treated rats were dosed b.i.d. for 7 consecutive days (from day 21–27) with 40 mg/kg of E-52862 (cumulative dose per day 80 mg/kg) or its solvent (0.5% HPMC). Mechanical sensitivity following repeated 7-day administration was assayed on day 28 before (pre-dose effect; washout period of approximately 16 hours after the last administration on day 27) and after additional dosing on day 28 (post-dose effect).

#### Chemotherapy-induced neuropathy after oxaliplatin (OX) treatment

Acquisition of neuropathy: Peripheral neuropathy was induced by repeated i.p. injections of OX (3 mg/kg, i.p.), an initial one and then 3 times a week for 2 weeks (7 injections; cumulative dose = 21 mg/kg, i.p.)^72^. Distilled water injections were used to reproduce the non-neuropathic condition in a control (baseline) group (Fig. 3A).

#### Evaluation of cold allodynia

Cold allodynia was assessed using the acetone test. In this test, the intensity of hind paw withdrawal was measured upon application of a drop of acetone to the plantar surface of both hind paws by using a score. Responses to acetone were graded to the following 4-point scale: 0 (no response), 1 (quick withdrawal, flick of the paw), 2 (prolonged withdrawal or marked flicking of the paw), 3 (repetitive flicking of the paw with licking or biting). The cumulative cold score is defined as the sum of the 6 scores for each rat, the minimum score being 0 (no response to any of the 6 trials) and the maximum score being 18 (repetitive flicking and licking or biting of paws on each of the six trials). Cold allodynia was assessed by measuring the responses to acetone on days 8, 15 and 16.

In order to assess the treatment effect of E-52862 on the expression of OX-induced neuropathic pain (“curative” protocol once pain has developed) (Fig. 3A), the compound was administered i.p. (20, 40 and 80 mg/kg) in a repeated dosing paradigm from day 8 until day 15, and its effect on cold allodynia was assessed on day 8 (first day of administration), day 15 (last day of administration) and after treatment completion on day 16. On testing days 8 and 15, E-52862 and vehicle (0.5% HPMC) were administered 30 min before the test. Gabapentin (100 mg/kg, p.o.) was used as a positive reference compound. Animals from the gabapentin-treated group were dosed 120 min before testing on day 8.

In order to assess a possible preventive effect of E-52862 on the development of OX-induced neuropathic pain (Fig. 4A), E-52862 was co-administered with OX. For this purpose, E-52862 was administered repeatedly b.i.d. at 40 mg/kg i.p., starting 2 days before the first injection of OX (3 mg/kg; i.p.) and throughout the OX treatment period (last OX injection on day 15). On the day of OX treatment, animals were dosed with the compound E-52862 30 min before OX injection. The acetone test was performed on days 8, 15 and 16 (24 h after the last administration).

### Statistical analysis

All data are presented as mean ± SEM. When several means were compared, all values were subjected to one-way analysis of variance (ANOVA) followed by post hoc Newman-Keuls test. To evaluate the development of diabetes, the comparison between baseline and post-STZ (day 4) glucose values was analyzed using the Student’s t-test. GraphPad Prism software (version 5.0; GraphPad Software Inc., La Jolla, CA, USA) was used. In all cases, the criterion for statistical significance was established at a *p* value less than 0.05.

## Additional Information

**How to cite this article**: Gris, G. *et al*. The selective sigma-1 receptor antagonist E-52862 attenuates neuropathic pain of different aetiology in rats. *Sci. Rep*. **6**, 24591; doi: 10.1038/srep24591 (2016).

## References

[b1] Baron R., Binder A. & Wasner G. Neuropathic pain: diagnosis, pathophysiological mechanisms, and treatment. Lancet Neurol. 9, 807–819 (2010). 20650402 10.1016/S1474-4422(10)70143-5

[b2] Kinloch R. A. & Cox P. J. New targets for neuropathic pain therapeutics. Expert. Opin. Ther. Targets. 9, 685–698 (2005). 16083337 10.1517/14728222.9.4.685

[b3] Burgess G. & Williams D. The discovery and development of analgesics: new mechanisms, new modalities. J. Clin. Invest. 120, 3753–3759 (2010). 21041957 10.1172/JCI43195 PMC2966322

[b4] Zamanillo D., Romero L., Merlos M. & Vela J. M. Sigma 1 receptor: a new therapeutic target for pain. Eur. J. Pharmacol. 716, 78–93 (2013). 23500210 10.1016/j.ejphar.2013.01.068

[b5] Drews E. & Zimmer A. Central sensitization needs sigma receptors. Pain 145, 69–270 (2009). 19577365 10.1016/j.pain.2009.06.016

[b6] Romero L. . Pharmacological properties of S1RA, a new sigma-1 receptor antagonist that inhibits neuropathic pain and activity-induced spinal sensitization. Br. J. Pharmacol. 166, 2289–2306 (2012). 22404321 10.1111/j.1476-5381.2012.01942.x PMC3448894

[b7] Latremoliere A. & Woolf C. J. Central sensitization: a generator of pain hypersensitivity by central neural plasticity. J. Pain 10, 895–926 (2009). 19712899 10.1016/j.jpain.2009.06.012 PMC2750819

[b8] Cendán C. M., Pujalte J. M., Portillo-Salido E. & Baeyens J. M. Antinociceptive effects of haloperidol and its metabolites in the formalin test in mice. Psychopharmacology (Berl). 182, 485–493 (2005a). 16075285 10.1007/s00213-005-0127-z

[b9] Cendán C. M., Pujalte J. M., Portillo-Salido E., Montoliu L. & Baeyens J. M. Formalin-induced pain is reduced in sigma(1) receptor knockout mice. Eur. J. Pharmacol. 511, 73–74 (2005b). 15777781 10.1016/j.ejphar.2005.01.036

[b10] Entrena J. M. . Antagonism by haloperidol and its metabolites of mechanical hypersensitivity induced by intraplantar capsaicin in mice: role of sigma-1 receptors. Psychopharmacology (Berl) 205, 21–33 (2009a). 19326101 10.1007/s00213-009-1513-8 PMC2695546

[b11] Entrena J. M. . Sigma-1 receptors are essential for capsaicin-induced mechanical hypersensitivity: studies with selective sigma-1 ligands and sigma-1 knockout mice. Pain 143, 252–261 (2009b). 19375855 10.1016/j.pain.2009.03.011

[b12] De la Puente B. . Sigma-1 receptors regulate activity-induced spinal sensitization and neuropathic pain after peripheral nerve injury. Pain 145, 294–303 (2009). 19505761 10.1016/j.pain.2009.05.013

[b13] Gris G., Merlos M., Vela J. M., Zamanillo D. & Portillo-Salido E. S1RA, a selective sigma-1 receptor antagonist, inhibits inflammatory pain in the carrageenan and complete Freund’s adjuvant models in mice. Behav. Pharmacol. 25, 226–235 (2014). 24776490 10.1097/FBP.0000000000000038

[b14] Tejada M. A. . Sigma-1 receptor inhibition reverses acute inflammatory hyperalgesia in mice: role of peripheral sigma-1 receptors. Psychopharmacology (Berl) 231, 3855–3869 (2014). 24639046 10.1007/s00213-014-3524-3

[b15] Nieto F. R. . Role of sigma-1 receptors in paclitaxel-induced neuropathic pain in mice. J. Pain 13, 1107–1121 (2012). 23063344 10.1016/j.jpain.2012.08.006

[b16] Abadias M., Escriche M., Vaqué A., Sust M. & Encina G. Safety, tolerability and pharmacokinetics of single and multiple doses of a novel sigma-1 receptor antagonist in three randomized phase I studies. Br. J. Clin. Pharmacol. 75, 103–117 (2013). 22607269 10.1111/j.1365-2125.2012.04333.x PMC3555050

[b17] Vos B. P., Strassman A. M. & Maciewicz R. J. Behavioral evidence of trigeminal neuropathic pain following chronic constriction injury to the rat’s infraorbital nerve. J. Neurosci. 14, 2708–2723 (1994). 8182437 10.1523/JNEUROSCI.14-05-02708.1994 PMC6577477

[b18] Ahlgren S. C. & Levine J. D. Mechanical hyperalgesia in streptozotocin-diabetic rats. Neuroscience 52, 1049–1055 (1993). 8450973 10.1016/0306-4522(93)90551-p

[b19] Ling B. . Behavioral and immunohistological assessment of painful neuropathy induced by a single oxaliplatin injection in the rat. Toxicology 234, 176–184 (2007). 17418472 10.1016/j.tox.2007.02.013

[b20] Obermann M. & Katsarava Z. Update on trigeminal neuralgia. Expert. Rev. Neurother. 9, 323–329 (2009). 19271941 10.1586/14737175.9.3.323

[b21] Pluijms W. . Evidence-based interventional pain medicine according to clinical diagnoses. 18. Painful diabetic polyneuropathy. Pain Pract. 11, 191–198 (2011). 21199315 10.1111/j.1533-2500.2010.00435.x

[b22] Farquhar-Smith P. Chemotherapy-induced neuropathic pain. Curr. Opin. Support Palliat. Care 5, 1–7 (2011). 21192267 10.1097/SPC.0b013e328342f9cc

[b23] Kayser V., Aubel B., Hamon M. & Bourgoin S. The antimigraine 5-HT 1B/1D receptor agonists, sumatriptan, zolmitriptan and dihydroergotamine, attenuate pain-related behaviour in a rat model of trigeminal neuropathic pain. Br. J. Pharmacol. 137, 1287–1297 (2002). 12466238 10.1038/sj.bjp.0704979 PMC1573605

[b24] Bennett G. J. Neuropathic pain in the orofacial region: clinical and research challenges. J. Orofac. Pain 18, 281–286 (2004). 15636009

[b25] Latrémolière A. . Differential implication of proinflammatory cytokine interleukin-6 in the development of cephalic versus extracephalic neuropathic pain in rats. J. Neurosci. 28, 8489–8501 (2008). 18716207 10.1523/JNEUROSCI.2552-08.2008 PMC6671060

[b26] Michot B., Bourgoin S., Viguier F., Hamon M. & Kayser V. Differential effects of calcitonin gene-related peptide receptor blockade by olcegepant on mechanical allodynia induced by ligation of the infraorbital nerve vs the sciatic nerve in the rat. Pain 153, 1939–1948 (2012). 22795918 10.1016/j.pain.2012.06.009

[b27] Li K. W., Kim D. S., Zaucke F. & Luo Z. D. Trigeminal nerve injury-induced thrombospondin-4 up-regulation contributes to orofacial neuropathic pain states in a rat model. Eur. J. Pain 18, 489–495 (2014). 24019258 10.1002/j.1532-2149.2013.00396.x PMC3947726

[b28] Colpaert F. C., Deseure K., Stinus L. & Adriaensen H. High-efficacy 5-hydroxytryptamine 1A receptor activation counteracts opioid hyperallodynia and affective conditioning. J. Pharmacol. Exp. Ther. 316, 892–899 (2006). 16254131 10.1124/jpet.105.095109

[b29] Christensen D., Gautron M., Guilbaud G. & Kayser V. Effect of gabapentin and lamotrigine on mechanical allodynia-like behaviour in a rat model of trigeminal neuropathic pain. Pain 93, 147–153 (2001). 11427326 10.1016/S0304-3959(01)00305-0

[b30] Deseure K., Koek W., Adriaensen H. & Colpaert F. C. Continuous administration of the 5-hydroxytryptamine1A agonist (3-Chloro-4-fluoro-phenyl)-[4-fluoro-4-[[(5-methyl-pyridin-2-ylmethyl) -amino]-methyl]piperidin-1-yl]-methadone (F 13640) attenuates allodynia-like behavior in a rat model of trigeminal neuropathic pain. J. Pharmacol. Exp. Ther. 306, 505–514 (2003). 12730352 10.1124/jpet.103.050286

[b31] Kwon Y. B., Jeong Y. C., Kwon J. K., Son J. S. & Kim K. W. The antinociceptive effect of sigma-1 receptor antagonist, BD1047, in a capsaicin induced headache model in rats. Korean J. Physiol. Pharmacol. 13, 425–429 (2009). 20054487 10.4196/kjpp.2009.13.6.425 PMC2802301

[b32] Roh D. H. & Yoon S. Y. Sigma-1 receptor antagonist, BD1047 reduces nociceptive responses and phosphorylation of p38 MAPK in mice orofacial formalin model. Biol. Pharm. Bull. 37, 145–151 (2014). 24152609 10.1248/bpb.b13-00690

[b33] Pyun K., Son J. S. & Kwon Y. B. Chronic activation of sigma-1 receptor evokes nociceptive activation of trigeminal nucleus caudalis in rats. Pharmacol. Biochem. Behav. 124, 278–283 (2014). 24992726 10.1016/j.pbb.2014.06.023

[b34] Gao F. & Zheng Z. M. Animal models of diabetic neuropathic pain. Exp. Clin. Endocrinol. Diabetes 122, 100–106 (2014). 24554509 10.1055/s-0033-1363234

[b35] Yamamoto H. . Pharmacological characterization of standard analgesics on mechanical allodynia in streptozotocin-induced diabetic rats. Neuropharmacology 57, 403–408 (2009). 19591853 10.1016/j.neuropharm.2009.06.037

[b36] Courteix C., Bardin M., Chantelauze C., Lavarenne J. & Eschalier A. Study of the sensitivity of the diabetes-induced pain model in rats to a range of analgesics. Pain 57, 153–160 (1994). 8090511 10.1016/0304-3959(94)90218-6

[b37] Aubel B. . Antihyperalgesic effects of cizolirtine in diabetic rats: behavioral and biochemical studies. Pain 110, 22–32 (2004). 15275748 10.1016/j.pain.2004.03.001

[b38] Gul H., Yildiz O., Dogrul A., Yesilyurt O. & Isimer A. The interaction between IL-1beta and morphine: possible mechanism of the deficiency of morphine-induced analgesia in diabetic mice. Pain 89, 39–45 (2000). 11113291 10.1016/S0304-3959(00)00343-2

[b39] Kamei J., Mizoguchi H., Narita M. & Tseng L. F. Therapeutic potential of PKC inhibitors in painful diabetic neuropathy. Expert. Opin. Investig. Drugs 10, 1653–1664 (2001). 10.1517/13543784.10.9.1653 11772275

[b40] Malcangio M. & Tomlinson D. R. A pharmacologic analysis of mechanical hyperalgesia in streptozotocin/diabetic rats. Pain 76, 151–157 (1998). 9696468 10.1016/s0304-3959(98)00037-2

[b41] Kiguchi S., Imamura T., Ichikawa K. & Kojima M. Oxcarbazepine antinociception in animals with inflammatory pain or painful diabetic neuropathy. Clin. Exp. Pharmacol. Physiol. 31, 57–64 (2004). 14756685 10.1111/j.1440-1681.2004.03950.x

[b42] Arnér S. & Meyerson B. A. Lack of analgesic effect of opioids on neuropathic and idiopathic forms of pain. Pain 33, 11–23 (1988). 2454440 10.1016/0304-3959(88)90198-4

[b43] Tesfaye S. & Selvarajah D. Advances in the epidemiology, pathogenesis and management of diabetic peripheral neuropathy. Diabetes. Metab. Res. Rev. 28 Suppl 1, 8–14 (2012). 22271716 10.1002/dmrr.2239

[b44] Moore R. A., Straube S., Wiffen P. J., Derry S. & McQuay H. J. Pregabalin for acute and chronic pain in adults. Cochrane Database Syst. Rev. 3, CD007076 (2009). 19588419 10.1002/14651858.CD007076.pub2 PMC4167351

[b45] Beyreuther B., Callizot N. & Stöhr T. Antinociceptive efficacy of lacosamide in a rat model for painful diabetic neuropathy. Eur. J. Pharmacol. 539, 64–70 (2006). 16682022 10.1016/j.ejphar.2006.04.009

[b46] Vela J. M., Merlos M. & Almansa C. Investigational sigma-1 receptor antagonists for the treatment of pain. Expert Opin. Investig. Drugs 24, 883–896 (2015). 10.1517/13543784.2015.1048334 26037209

[b47] Weickhardt A., Wells K. & Messersmith W. Oxaliplatin-induced neuropathy in colorectal cancer. J. Oncol. 2011, 201593 (2011). 22203844 10.1155/2011/201593 PMC3238400

[b48] Jaggi A. S. & Singh N. Mechanisms in cancer-chemotherapeutic drugs-induced peripheral neuropathy. Toxicology 291, 1–9 (2012). 22079234 10.1016/j.tox.2011.10.019

[b49] Flatters S. J. & Bennett G. J. Ethosuximide reverses paclitaxel- and vincristine-induced painful peripheral neuropathy. Pain 109, 150–61 (2004). 15082137 10.1016/j.pain.2004.01.029

[b50] Gauchan P. . Mechanical allodynia induced by paclitaxel, oxaliplatin and vincristine: different effectiveness of gabapentin and different expression of voltage-dependent calcium channel alpha(2)delta-1 subunit. Biol. Pharm. Bull. 32, 732–734 (2009). 19336915 10.1248/bpb.32.732

[b51] Sakurai M. . Oxaliplatin-induced neuropathy in the rat: involvement of oxalate in cold hyperalgesia but not mechanical allodynia. Pain 147, 165–174 (2009). 19782472 10.1016/j.pain.2009.09.003

[b52] Fehrenbacher J. C., Taylor C. P. & Vasko M. R. Pregabalin and gabapentin reduce release of substance P and CGRP from rat spinal tissues only after inflammation or activation of protein kinase C. Pain 105, 133–141 (2003). 14499429 10.1016/s0304-3959(03)00173-8

[b53] Nieto F. R. . Genetic inactivation and pharmacological blockade of sigma-1 receptors prevent paclitaxel-induced sensory-nerve mitochondrial abnormalities and neuropathic pain in mice. Mol. Pain 10, 11 (2014). 24517272 10.1186/1744-8069-10-11 PMC3924235

[b54] Dickenson A. H. & Sullivan A. F. Evidence for a role of the NMDA receptor in the frequency dependent potentiation of deep rat dorsal horn nociceptive neurones following C fibre stimulation. Neuropharmacology 26, 1235–1238 (1987). 2821443 10.1016/0028-3908(87)90275-9

[b55] Li J., Simone D. A. & Larson A. A. Windup leads to characteristics of central sensitization. Pain 79, 75–82 (1999). 9928779 10.1016/S0304-3959(98)00154-7

[b56] Herrero J. F., Laird J. M. & López-García J. A. Wind-up of spinal cord neurones and pain sensation: much ado about something? Prog. Neurobiol. 61, 169–203 (2000). 10704997 10.1016/s0301-0082(99)00051-9

[b57] Mazo I. . Effects of centrally acting analgesics on spinal segmental reflexes and wind-up. Eur. J. Pain 19, 1012–1020 (2014). 25469831 10.1002/ejp.629

[b58] Woolf C. J. & Thompson S. W. The induction and maintenance of central sensitization is dependent on N-methyl-D-aspartic acid receptor activation; implications for the treatment of post-injury pain hypersensitivity states. Pain 44, 293–299 (1991). 1828878 10.1016/0304-3959(91)90100-C

[b59] Monnet F. P., Debonnel G., Junien J. L. & De Montigny C. N-methyl-D-aspartate-induced neuronal activation is selectively modulated by sigma receptors. Eur. J. Pharmacol. 179, 441–445 (1990). 2163857 10.1016/0014-2999(90)90186-a

[b60] Bergeron R., de Montigny C. & Debonnel G. Potentiation of neuronal NMDA response induced by dehydroepiandrosterone and its suppression by progesterone: effects mediated via sigma receptors. J. Neurosci. 16, 1193–1202 (1996). 8558248 10.1523/JNEUROSCI.16-03-01193.1996 PMC6578822

[b61] Martina M., Turcotte M. E., Halman S. & Bergeron R. The sigma-1 receptor modulates NMDA receptor synaptic transmission and plasticity via SK channels in rat hippocampus. J. Physiol. 578, 143–57 (2007). 17068104 10.1113/jphysiol.2006.116178 PMC2075134

[b62] Roh D. H. . Sigma-1 receptor-induced increase in murine spinal NR1 phosphorylation is mediated by the PKCalpha and epsilon, but not the PKCzeta, isoforms. Neurosci. Lett. 477, 95–99 (2010). 20417251 10.1016/j.neulet.2010.04.041

[b63] Kim H. W. . Intrathecal treatment with sigma1 receptor antagonists reduces formalin-induced phosphorylation of NMDA receptor subunit 1 and the second phase of formalin test in mice. Br. J. Pharmacol. 148, 490–498 (2006). 16682960 10.1038/sj.bjp.0706764 PMC1751783

[b64] Roh D. H. . Intrathecal injection of the sigma(1) receptor antagonist BD1047 blocks both mechanical allodynia and increases in spinal NR1 expression during the induction phase of rodent neuropathic pain. Anesthesiology 109, 879–89 (2008). 18946301 10.1097/ALN.0b013e3181895a83

[b65] Rodríguez-Muñoz M. . The σ1 receptor engages the redox-regulated HINT1 protein to bring opioid analgesia under NMDA receptor negative control. Antioxid. Redox Signal. 22, 799–818 (2015). 25557043 10.1089/ars.2014.5993 PMC4367239

[b66] Zimmermann M. Ethical guidelines for investigations of experimental pain in conscious animals. Pain 16, 109–110 (1983). 6877845 10.1016/0304-3959(83)90201-4

[b67] Vidal-Torres A. . Effects of the selective sigma-1 receptor antagonist S1RA on formalin-induced pain behavior and neurotransmitter release in the spinal cord in rats. J. Neurochem. 129, 484–494 (2014). 24384038 10.1111/jnc.12648

[b68] Gregg J. M. A surgical approach to the ophthalmic-maxillary nerve trunks in the rat. J. Dent. Res. 52, 392 (1973). 4511325 10.1177/00220345730520024001

[b69] Jacquin M. F. & Zeigler H. P. Trigeminal orosensation and ingestive behavior in the rat. Behav. Neurosci. 97, 62–97 (1983). 6838727 10.1037//0735-7044.97.1.62

[b70] Bennett G. J. & Xie Y. K. A peripheral mononeuropathy in rat that produces disorders of pain sensation like those seen in man. Pain 33, 87–107 (1988). 2837713 10.1016/0304-3959(88)90209-6

[b71] Randall L. O. & Selitto J. J. A method for measurement of analgesic activity on inflamed tissue. Arch. Int. Pharmacodyn. Ther. 111, 409–419 (1957). 13471093

[b72] Polomano R. C., Mannes A. J., Clark U. S. & Bennett G. J. A painful peripheral neuropathy in the rat produced by the chemotherapeutic drug, paclitaxel. Pain 94, 293–304 (2001). 11731066 10.1016/S0304-3959(01)00363-3

